# Advanced glycation end products induce brain-derived neurotrophic factor release from human platelets through the Src-family kinase activation

**DOI:** 10.1186/s12933-017-0505-y

**Published:** 2017-02-08

**Authors:** Kazuo Furukawa, Ichiro Fuse, Yuriko Iwakura, Hidekazu Sotoyama, Osamu Hanyu, Hiroyuki Nawa, Hirohito Sone, Nobuyuki Takei

**Affiliations:** 1 Department of Molecular Neurobiology, Brain Research Institute, Niigata University, Asahimachi, Niigata, 951-8585 Japan; 2 Department of Hematology, Endocrinology and Metabolism, Niigata University Faculty of Medicine, Niigata, Japan; 3 Japanese Red Cross Niigata Blood Center, Niigata, Japan

**Keywords:** AGE, BDNF, Platelets, Release, Ca^2+^, Src family kinases, Syk

## Abstract

**Background:**

Brain-derived neurotrophic factor (BDNF) exerts beneficial effects not only on diabetic neuropathies but also on cardiovascular injury. There is argument regarding the levels of serum BDNF in patients with diabetes mellitus (DM). Because BDNF in peripheral blood is rich in platelets, this may represent dysregulation of BDNF release from platelets. Here we focused on advanced glycation end products (AGEs), which are elevated in patients with DM and have adverse effects on cardiovascular functions. The aim of this study is to elucidate the role of AGEs in the regulation of BDNF release from human platelets.

**Methods:**

Platelets collected from peripheral blood of healthy volunteers were incubated with various concentrations of AGE (glycated-BSA) at 37 °C for 5 min with or without BAPTA-AM, a cell permeable Ca^2+^ chelator, or PP2, a potent inhibitor of Src family kinases (SFKs). Released and cellular BDNF were measured by ELISA and calculated. Phosphorylation of Src and Syk, a downstream kinase of SFKs, in stimulated platelets was examined by Western blotting and immunoprecipitation.

**Results:**

AGE induced BDNF release from human platelets in a dose-dependent manner, which was dependent on intracellular Ca^2+^ and SFKs. We found that AGE induced phosphorylation of Src and Syk.

**Conclusions:**

AGE induces BDNF release from human platelets through the activation of the Src-Syk-(possibly phospholipase C)-Ca^2+^ pathway. Considering the toxic action of AGEs and the protective roles of BDNF, it can be hypothesized that AGE-induced BDNF release is a biological defense system in the early phase of diabetes. Chronic elevation of AGEs may induce depletion or downregulation of BDNF in platelets during the progression of DM.

**Electronic supplementary material:**

The online version of this article (doi:10.1186/s12933-017-0505-y) contains supplementary material, which is available to authorized users.

## Background

Brain-derived neurotrophic factor (BDNF) is a member of the neurotrophin family and plays essential roles in differentiation and survival of central and peripheral neurons [1]. Thus, BDNF has been implicated in neurodegenerative diseases and has been tried as a therapeutic intervention for such diseases [2]. In addition to these classic neurotrophic actions, BDNF regulates synaptic plasticity in the brain [3, 4]. Because of its effects on functional alterations in the central nervous system, it was implicated in psychiatric diseases such as depression [5]. Another face of BDNF has been emerging, which is the regulation of food intake and energy metabolism [6, 7]. The involvement of BDNF in diabetes has been studied from those aspects [8].

Diabetes mellitus (DM) is associated with macrovascular complications, including increased risks of coronary heart disease, stroke and amputation, and microvascular complications, such as nephropathy, retinopathy and neuropathy. In addition to its beneficial effects on neuropathies [9] and retinopathies [10], the cardiovascular protective effects of BDNF were reported. BDNF was shown to prevent or ameliorate myocardial infraction [11] and to promote revascularization after ischemic injury [12]. Furthermore, the association of blood BDNF levels with cardiovascular diseases, such as angina pectoris [13] and heart failure [14, 15] were reported, with lower levels of BDNF being associated with the risk of cardiovascular diseases.

These effects must come from outside of the CNS, presumably from BDNF in blood. Both increased [16, 17] and decreased [18–20] BDNF were reported in blood of patients with DM. The mechanism of this inconsistency remains unclear although blood glucose levels, duration of DM, medications, gender, etc., may play a role.

Platelets are the major source of BDNF in human blood, at least under physiological conditions [21, 22], which may indicate that serum BDNF levels reflect the amount released from platelets. Although the causality and mechanisms remain unclear, it is possible that the dynamics of BDNF release are dysregulated in diabetes patients, thus altering serum levels. Therefore, we investigated the acute BDNF release from platelets from healthy individuals. BDNF is known to be released from platelets in response to certain stimulants such as thrombin [22]. Thus, we examined the effects of molecules that are abundant in the blood of DM patients and whose receptors are expressed in platelets.

We focused on advanced glycation end products (AGEs). AGEs are a group of carbonyl compounds produced by the non-enzymatic reaction of reducing sugars and amino groups of proteins, lipids and nucleic acids, which is called the Maillard reaction [23]. AGE levels are high in blood of patients with diabetes because of their chronic hyperglycemia [23], and AGE accumulation induces adverse effects on endothelial cells [24] and cardiovascular systems [25]. Their receptors, such as receptors for AGE (RAGE) [26], CD36 [27] and 5HT2A/C receptor [28], are expressed in human platelets.

To analyze the dynamics of acute BDNF release from platelets under physiological conditions, we examined the effect of AGEs on BDNF release from platelets of healthy control participants (non-diabetic) and analyzed their signaling mechanisms.

## Methods

### Materials

Glycated-BSA, used as the AGE in this study, was purchased from Bio Vision. Human thrombin was obtained from Sigma-Aldrich. Prostaglandin E1 (PGE1), anti-c-Src and anti-phospho-Src antibodies were purchased from Santa Cruz Biotechnology. BAPTA-AM and PP2 were obtained from Dojin and Abcam, respectively. Anti-Syk antibody and anti-phosphotyrosine antibody (PY100) were purchased from Cell Signaling Technology.

### Preparation of platelet samples, serum and whole blood

The Ethics Committee of Niigata University approved the study and informed consent was obtained from participants. Blood was collected from healthy volunteers aged 31–53 years according to the method described previously [29]. Briefly, venous blood was collected using a 22 gauge needle and was transferred to a tube containing 3.2% sodium citrate. Platelet-rich plasma (PRP) was obtained by centrifugation at 200×*g* for 10 min at room temperature. Acid citrate dextrose solution (ACD) containing PGE1 (1 μM) was added to PRP at a final concentration of 15% and then washed twice with Tris/EDTA/saline buffer (10 mM Tris, 1 mM EDTA, 150 mM NaCl, pH 7.5) containing PGE1. Platelets were finally diluted in Ca^2+^ -free tyrode buffer (138 mM NaCl, 2.7 mM KCI, 1 mM MgCl_2_, 3 mM NaH_2_PO_4_, 5 mM glucose, 10 mM HEPES, pH 7.4). Platelet samples (1 × 10^8^ cells/500 μl) were incubated with the indicated agents at 37 °C for 5 min.

BAPTA-AM or PP2 was added 5 min prior to the AGE stimulation. Platelet activation was stopped by adding cold EDTA solution (1 mM) and the mixture was immediately centrifuged at 800×*g* for 10 min. The supernatants were collected and the residual pellet was lysed with lysis buffer (20 mM NaH_2_PO_4_ 2 mM 2 mM NaH_2_PO_4_, 150 mM NaCl 0.5% Triton-X100: pH 7.5) containing protease and phosphatase inhibitor cocktails (cOmplete and PhosStop, Roche). All procedures using platelets were performed under sterile conditions.

Whole blood samples were prepared by dissolving them in lysis buffer and sonication. After centrifugation at 15,000×*g* for 5 min, the supernatant was collected. Serum was prepared by incubating blood at 37 °C for 1 h and centrifuging at 800×*g* for 10 min. The supernatants were collected as serum samples. All samples were stored at −80 °C until use.

### Measurements of BDNF, PF4 and 5-HT

The concentrations of BDNF in serum, whole blood, platelet supernatant and platelet lysate were measured by BDNF ELISA according to the reported protocol [30]. Quantitative assays for platelet factor 4 (PF4) were performed by ELISA according to the manufacturer’s instructions (R&D). 5-hydroxytryptamine(5-HT) was measured by high performance liquid chromatography with electrochemical detection as described Previously [31].

### Calculation of released molecules from platelets

The levels of released BDNF, PF4 and 5-HT from platelets were represented as percentages of their content in platelets according to the following formula: percentage of release = (amount released from agonist-stimulated platelets)/(amount released from agonist- stimulated platelets + amount of platelet lysate) × 100.

### Western blot analysis and immunoprecipitation

Samples for Western blotting and immunoprecipitation were prepared under the same conditions as for the release assay, and analyses were performed essentially as reported [32]. Briefly, platelets (5 × 10^8^ cells/300 μl) were lysed and sonicated in sample buffer (10 mM Tris–HCl, 150 mM NaCl, 2% SDS, cOmplete, PhosStop). Equal amounts of protein were subjected to 10% sodium dodecyl sulfate-polyacrylamide gel electrophoresis and transferred to PVDF membranes. The membranes were incubated with primary antibodies and then with secondary antibodies (DAKO; 1:10,000). Peroxidase activity was visualized with a chemiluminescent reagent (Immunostar: Wako) and G:Box apparatus (Syngene). Quantification was performed by GeneSys software (Syngene). For immunoprecipitation [32], platelets (5 × 10^8^ cells/300 μl) were lysed and sonicated in RIPA buffer (50 mM Tris/HCI, 150 mM NaCI, 1 mM EDTA, 5 mM EGTA, 1% NP-40, 20 mM glycerophosphate, 0.5 M DTT) containing cOmplete and PhosStop. After centrifugation, supernatants were preabsorbed with protein G-Sepharose (GE Healthcare). Supernatants were then incubated with anti-Syk antibodies overnight at 4 °C and the immunocomplexes were precipitated by the addition of 40 μl of Protein GSepharose for 2 h. After brief centrifugation, immunoprecipitates were washed 3 times with RIPA buffer and used for Western blotting.

### Statistical analysis

Data are expressed throughout as mean ± SEM. Statistical significance was determined using the Student’s *t* test for comparisons of two groups and one-way or two-way ANOVA for multiple comparisons. Tukey’s test was used for post hoc comparison when the F value was significant (p < 0.05).

## Results

### Levels of BDNF had large individual differences but the ratio of release was constant

The levels of BDNF in whole blood, serum and platelets from individuals as measured by ELISA are shown in Additional file 1: Figure S1. Ten healthy volunteers (8 men and 2 women) were examined. Mean age was 38.8 ± 2.4 years and mean blood glucose was 106 ± 12.2 mg/dl (data were presented as mean ± SD). Other information on blood data is provided in Additional file 2: Table S1. None of the blood indices were correlated with BDNF content in whole blood, serum or platelets.

**Fig. 1:**
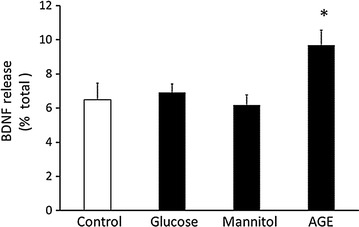
AGE, but not high glucose and high mannitol, induced BDNF release from platelets. Each platelet sample (1 × 10^8^ cells/tube) was incubated with Ca^2+^-free tyrode buffer containing 0.1% BSA (C, control), 25 mM glucose (G), 25 mM mannitol (M) and 100 μg/ml AGE (A) for 5 min at 37 °C (n = 8). Data are presented as the ratio of released BDNF/(released BDNF + BDNF content) (mean ± SEM). Statistical analysis was performed by one-way ANOVA. *p < 0.05 vs. control

Although the contents in each fraction varied greatly among participants, the ratio of BDNF release from platelets was rather constant as shown in Fig. 1 (see also Additional file 3: Figure S2). Thus, BDNF release in response to stimulation in different individuals was analyzed in this study.

Before the main experiments, the assay system was validated by using thrombin, previously reported as a secretagogue of BDNF from platelets, as a positive control. As reported previously [22], thrombin strongly induced BDNF release (control: 30.2 + 24.4 pg/100 μl vs. 0.1 U/ml thrombin 321 + 192.9 pg/100 μl).

### AGE, not high glucose or mannitol, induced BDNF release from human platelets

**Fig. 2:**
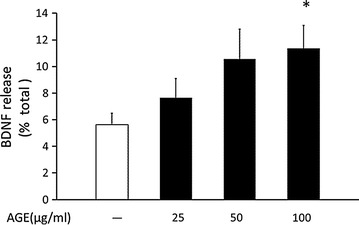
AGE induced BDNF release from platelets in a dose-dependent manner. After platelet samples (1 × 10^8^ cells/tube) were incubated at 37 °C by stimulations with 0.1% BSA (control), 25, 50 and 100 μg/ml AGE for 5 min, the supernatants and lysates were collected and BDNF contents were measured by ELISA (n = 10). Data were presented as the ratio of released BDNF/(released BDNF + BDNF content) (mean ± SEM). Statistical analysis was performed by one-way ANOVA. *p < 0.05 vs. control

Since the validity of the assay system was guaranteed, the effects of high glucose, mannitol and AGE on BDNF release from platelets were examined because hyperglycemia is the primary manifestation of diabetes. We used mannitol as the osmolarity control and AGE as a representative of chronic hyperglycemia. Neither high glucose nor mannitol but AGE, glycated-BSA in this study, significantly induced BDNF release (Fig. 1). The action of AGE on BDNF release was dose-dependent (Fig. 2) and was saturated at a dose of 100 μg/ml up to 1 mg/ml (Additional file 3: Figure S2). The effect of AGE was rather acute (5 min) (Fig. 1). Although AGE tended to increase BDNF release even at 20 and 60 min, a significant increase was only observed at 5 min after stimulation (Additional file 4: Figure S3). AGE, even at the maximum dose we used in this study, did not have an acute toxic effect on platelets.

To further analyze the role of AGE as a secretion-inducing substance for platelets, the release of PF4 and 5-HT, contained in alpha- and dense granule vesicles, respectively, was examined. Whereas AGE induced PF4 release in a manner similar to BDNF release, it had little effect on 5-HT release, although the effect was significant (see Additional file 5: Figure S4). The results suggest that the AGE-evoked release under this assay condition was rather specific for the alpha granule component.

### AGE-induced BDNF release was intracellular Ca^2+^ -dependent

A variety of agonists induce the elevation of cytosolic Ca2 + in platelets by enhancing the release of Ca^2+^ from the intracellular store, an event that plays a crucial role in platelet function [33]. AGE has been reported to increase cytosolic Ca^2+^ in mouse cardiomyocytes [34] and human platelets [35]. Thus, the Ca^2+^ -dependency of BDNF release was examined. Because the assay buffer did not contain Ca^2+^ (extracellular Ca^2+^ -free condition), the role of intracellular Ca^2+^ on AGE-induced BDNF release was analyzed using BAPTA-AM, a cell permeable Ca^2+^ chelator. The concentration of BAPTA-AM (10 μM) was determined according to previous reports [36, 37].

**Fig. 3:**
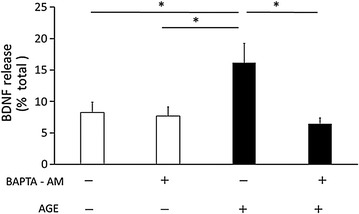
BAPTA-AM completely inhibited AGE-induced BDNF release from platelets. Platelet samples (1 × 10^8^ cells/tube) were pretreated with or without 10 μM BAPTA-AM for 5 min, then 100 μg/ml AGE was added to the samples for 5 min. Data were presented as the ratio of released BDNF/(released BDNF + BDNF content) (mean ± SEM). Statistical analyses were performed by SPSS two-way ANOVA with post hoc test. *White bars* control (0.1% BSA), *black bars* AGE (100 μg/ml). *p < 0.05 for indicated comparisons

BAPTA-AM completely abolished the effect of AGE on BDNE release (Fig. 3), indicating that intracellular Ca^2+^ is essential for this phenomenon. We also confirmed that AGE (100 μg/ml) increased intracellular Ca^2+^ levels at 3 min after stimulation by using Oregon Green (Thermo Fisher) indicator (Additional file 6: Figure S5).

### AGE-induced BDNF release was dependent on Src family kinases (SFKs)

**Fig. 4:**
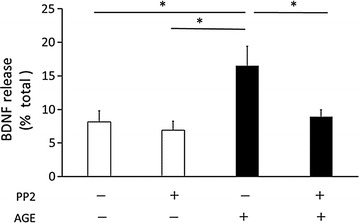
PP2 completely inhibited AGE-induced BDNF release from platelets. Platelets (1 × 10^8^ cells/tube) were pretreated with or without 10 μM PP2 for 5 min, then 100 μg/ml AGE was added to the samples for 5 min. Data were presented as the ratio of released BDNF/(released BDNF + BDNF content) (mean ± SEM). Statistical analyses were performed by SPSS two-way ANOVA with post hoc test. *White bars* control (0.1% BSA), *black bars* AGE (100 μg/ml). In this *figure*, an interaction between AGE-PP2 was not detected. *p < 0.05 for indicated comparisons

To further analyze the signaling mechanism, involvement of the SFKs was examined. The reasons for this analysis were that (1) platelets express a high level of Src (0.2–0.4% of total proteins) [38], (2) AGEs activate Src [39], (3) SFKs physically interact with CD36 [40] and 4) SFKs are linked to the increase in intracellular Ca^2+^ through the Syk-phospholipase Ca^2+^—IP3 pathway [41]. Thus, the effects of PP2, a potent inhibitor of SFKs, were examined. PP2 completely inhibited the effect of AGE on BDNF release (Fig. 4), while it had a moderate suppressive effect on thrombin-induced release (thrombin: 46.9 ± 5.2 vs. thrombin + PP2: 42.7 ± 13.0; an approximately 0.9% reduction).

**Fig. 5:**
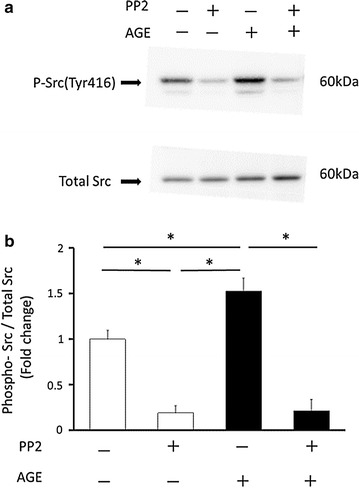
AGE induces Src phosphorylation in platelets. **a** Western blot analysis of phosphor-Src (Tyr416) and total c-Src after a 5-min stimulation of 100 μg/ml AGE with or without 10 μM PP2. **b** The *graph* shows quantification of the P-Src/Total Src ratio. Data are presented as fold changes compared to the control (n = 4, mean ± SEM). *White bars* control, *black bars* AGE (100 μg/ml). Statistical analysis was performed by SPSS two-way ANOVA with post hoc test (n = 4). *p < 0.05 for indicated comparisons

**Fig. 6:**
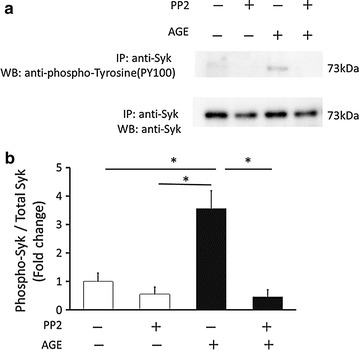
AGE induced Syk phosphorylation in platelets. **a** Immunoprecipitation-western blot analysis of Syk phosphorylation after a 5-min stimulation of 100 μg/ml AGE with or without 10 μM PP2. Samples were immunoprecipitated with and Syk antibody, then western blotting with anti-phospho tyrosine antibody (PY100). **b** The *graph* shows quantification of the P-Syk/Total Syk ratio. Data are presented as fold changes compared to the control (n = 4, mean ± SEM). Statistical analyses were performed by SPSS two-way ANOVA with post hoc test. *p < 0.05 for indicated comparisons

To confirm whether AGE actually activates Src in human platelets, Western blotting was performed. As expected, AGE rapidly induced the phosphorylation of Src at Tyr416 and that action was blocked by PP2 (Fig. 5). Furthermore, phosphorylation of Syk, a downstream kinase of SFKs, was also induced by AGE and inhibited by PP2 as revealed by immunoprecipitation-Western blotting analysis (Fig. 6). Those results suggest that AGE activates SFKs and then activates Syk to increase intracellular Ca^2+^ .

## Discussion

Reports of serum BDNF levels in patients with DM have been controversial. In comparison with non-diabetic individuals, some studies found that serum BDNF levels in patients with DM were lower [18–20] while others found higher values [16, 17].

Because the major origin of BDNF in blood is platelets [21, 22 also see Additional file 1: Figure S1, Additional file 2: Table S1], alterations in BDNF levels may be the result of dysregulation of BDNF release from platelets. Therefore, we examined the acute effects of high glucose, a primary indication of DM, and AGE, which are resultant products of chronic hyperglycemia.

Here, we revealed that AGE, a glycated-BSA in this study, induced BDNF release from human platelets through intracellular Ca^2+^ elevation possibly via the Src-Syk pathway.

AGEs are heterogeneous carbonyl compounds that are formed by the Maillard reaction between reducing sugars and the amino groups of protein, lipids and nucleic acids. Because of the chronic hyperglycemia in diabetic patients, AGE accumulation in blood is accelerated in those individual [42]. AGE accumulation correlated with microvascular lesions that cause diabetic retinopathy [43] or nephropathy [44]. In addition, AGE was reported to enhance aggregation and activation of platelets [35]. These abnormal platelets may be related to the risk of the development of cardiovascular complications [45]. Results of a clinical study also supported the concept that high AGE in diabetes mellitus is related to the risk of peripheral arterial diseases [46].

The deleterious action on endothelial cells by AGEs is mediated by their receptors. While many receptors for AGEs have been identified, the receptor for AGEs (RAGE) is the most characterized. Upon binding of AGEs to the RAGE, a variety of downstream responses occur, such as production of reactive oxygen species and expression of cytokines, cell adhesion molecules, etc. that lead to cellular insults [47]. While the expression of RAGE [26] as well as CD36 [27] and 5HT2A/C receptor [28] were reported on the platelet surface, it remains which (or all) receptors are responsive to AGE for BDNF release.

BDNF is a neurotrophic factor promoting differentiation and survival and modulating synaptic plasticity in central and peripheral neurons through its cognate receptor TrkB. Outside of the nervous system, TrkB expression was reported in several cell types such as immune [48], pancreatic alpha [49], endothelial [50] and myocardial [51] cells. In contrast to AGEs, BDNF was shown to exert a protective action on these cells [12, 52]. Exogenous BDNF induced vasodilatation and protected against vascular injury and thrombus formation in the walls of cerebral arteries [53] and also was noted to act on revascularization [12]. Clinical observations showed that low blood BDNF levels are associated with a high risk of heart failure [14, 15, 54]. Thus, regulated release of BDNF from platelets may be an important mechanism for maintaining cardiovascular homeostasis.

Mechanism of BDNF release has been well characterized in neuronal cells. BDNF is released in an activity‒dependent manner; in other words, depolarization-induced Ca^2+^ influx triggers its release [55]. Other studies showed that an increase in cytosolic Ca^2+^ derived from intracellular Ca^2+^ stores is sufficient for BDNF release [56]. These results indicate that the increase in intracellular Ca^2+^, whether it comes from an extracellular space or intracellular stores, induces BDNF release. Thus, Ca^2+^-dependency of BDNF release from platelets was examined. In the present study, Ca^2+^ ions were not included in the assay buffer to avoid basal platelet activation. Ca^2+^-dependency was analyzed by using BAPTA-AM, a cell permeable Ca^2+^-chelator. BAPTA-AM completely inhibited AGE-induced BDNF release, suggesting that the BDNF release mechanism is rather common among different cell types.

Similar to other growth factors, BDNF is contained in α-granules of platelets [57]. To determine the action of AGE on alpha-granules, release of another molecule in these granules was examined. AGE induced the release of PF4, one of the contents of alpha-granules. Although AGE significantly increased the release of 5-HT in dense granules, the ratio to its total content was quite low (Additional file 3: Figure S2). This suggests that under extracellular Ca^2+^-free conditions, 5-HT release is not sufficiently driven by a particular stimulation.

How is Ca^2+^ released from intracellular stores? We examined the roles of SFKs because a previous study indicated that there were high levels of Src in platelets [38] and that SFKs were involved in in AGE-induced signaling. In addition, activation of SFKs was shown to lead to an increase in intracellular Ca^2+^ [41, 58]. AGE was reported to induce Src activation in vascular endothelial cells through RAGE [39]. CD36, another receptor for AGE, interacted with Fyn, Lyn and Yes, members of SFKs in platelets [40]. Thus, whether the inhibitor of kinases suppresses the effect of AGE was analyzed. PP2, a potent Src family kinase inhibitor, completely blocked the AGE-induced BDNF release. The results suggest that Src and/or other members of the family kinases are activated by AGE in human platelets. Indeed, western blot analysis using phospho-Src antibody showed that AGE increased the phosphorylation of Src (Fig. 5). Although a comprehensive investigation is awaited, other members of SFKs may be involved in the process of BDNF release. In B-cells, Lyn, a member of SFKs, phosphorylates and activates Syk, which induces Ca^2+^ release from the stores through phospholipase Cγ (PLCγ)-IP3 [41]. Syk was shown to be activated in platelets and increase intracellular Ca^2+^ in response to stimuli [58]. AGE actually induced phosphorylation of Syk (Fig. 6). It was inhibited by PP2, suggesting that it occurred downstream of SFKs after AGE stimulation. Thus, the results obtained in this study suggest that AGE activates SFKs, at least Src, and that downstream Syk then increases intracellular Ca^2+^ possibly through PLCγ-IP3 activation.

Considering the toxic action of AGEs and protective roles of BDNF, it can be hypothesized that AGE-induced BDNF release is a biological defense system in the early phase of diabetes when the levels of AGEs are becoming higher. To protect against AGE-induced damage to, for example, vascular endothelial cells, platelets release BDNF to protect these cells in an early phase of the disease. However, a chronic elevation of AGEs may induce depletion or downregulation of BDNF in platelets during the progression of DM. Actually, a recent report supported this idea. While the serum BDNF levels are higher in prediabetic or early diabetic individuals than in normoglycemic persons, the serum BDNF levels are lower in patients with longstanding DM than in normal controls [16]. Thus, at least one causative factor in the breakdown of serum BDNF homeostasis in DM patients seems to be due to the accumulation of AGEs in the blood.

## Conclusions

AGE induces BDNF release from human platelets through the activation of the SrcSyk-(possibly phospholipase C)-Ca^2+^ pathway. Considering the toxic action of AGEs and the protective roles of BDNF, it can be hypothesized that AGE-induced BDNF release is a biological defense system in the early phase of diabetes. Chronic elevation of AGEs may induce depletion or downregulation of BDNF in platelets during the progression of DM.

## Additional file

**Additional file 1: Figure S1.** Scatter plot of BDNF levels in whole blood, serum and platelets (1 × 10^8^ cells) (n = 10). **Table S1.** The blood information of the volunteers. The blood samples were sent to SRL Inc, Japan. and fasting plasma glucose (FPG), fasting immunoreactive insulin (FIRI), HbA1c was analyzed at SRL. The homeostasis model assessment of insulin resistance (HOMA-IR) was calculated from FIRI and FPG by the following equation: HOMA-R = FIRI (mU/l) × FPG (mmol/l)/22.5. Data are means ± SEM. **Figure S2.** BDNF release at higher AGE concentrations (100 μg–1 mg/ml AGE). Data are presented as means ± SEM (n = 8). Statistical analyses were performed by SPSS one-way ANOVA. *p < 0.05 vs control. Note that 100 mg/ml was the saturation dose. **Figure S3.** Time course of AGE-induced BDNF release. BDNF release was measured at 5, 20 and 60 min after AGE stimulation. White bar, control; light grey bar, 25 μg/ml AGE; dark grey bar, 50 μg/ml AGE; black bar, 100 μg/ml AGE (n = 8, means ± SEM). Statistical analysis was performed by one-way ANOVA. *p < 0.05 vs control. The graph at 5 min is same as Fig. 2. **Figure S4.** AGE induced PF4 and 5-HT release from human platelets. Data were presented as means ± SEM (n = 8). Statistical analysis were performed by SPSS paired t test. White bars, control; black bars, AGE (100 μg/ml). *p < 0.05 vs control. **Figure S5.** AGE increased intracellular Ca^2+^ levels in platelets. Platelet was incubated with Oregon Green 488 (2 mM, Thermo Fisher) for 30 min and was washed with assay buffer (same as BDNF assay buffer). (a), Fluorescent intensity was measured by fluorescent microplate reader (Ex: 490 nm, EM: 530 nM) 3 min after AGE stimulation according to the manufacture’s instruction. Data are presented as means ± SEM (n = 30 (well)). Typical fluorescent photomicrographs of control (b) and AGE-treated (c) platelets.

## Abbreviations

AGE: advanced glycation end product
BDNF: brain-derived neurotrophic factor
DM: diabetes mellitus
5-HT: 5-Hydroxytryptamine
IP3: inositol trisphosphate
PF4: platelet factor 4
PLC γ: phospholipase C γ
SFKs: Src family kinases
TrkB: tropomyosin-related kinase B
