# Supplementary material for: Advanced glycation end products induce brain-derived neurotrophic factor release from human platelets through the Src-family kinase activation
Source: Cardiovasc Diabetol. 2017 Feb 8;16:20. doi: 10.1186/s12933-017-0505-y (PMC5299653; doi:10.1186/s12933-017-0505-y)
Supplement: Supplementary file 1 — Additional file 1: Figure S1. Scatter plot of BDNF levels in whole blood, serum and platelets (1 × 10^8^ cells) (n = 10). Table S1. The blood information of the volunteers. The blood samples were sent to SRL Inc, Japan. and fasting plasma glucose (FPG), fasting immunoreactive insulin (FIRI), HbA1c was analyzed at SRL. The homeostasis model assessment of insulin resistance (HOMA-IR) was calculated from FIRI and FPG by the following equation: HOMA-R = FIRI (mU/l) × FPG (mmol/l)/22.5. Data are means ± SEM. Figure S2. BDNF release at higher AGE concentrations (100 μg–1 mg/ml AGE). Data are presented as means ± SEM (n = 8). Statistical analyses were performed by SPSS one-way ANOVA. *p < 0.05 vs control. Note that 100 mg/ml was the saturation dose. Figure S3. Time course of AGE-induced BDNF release. BDNF release was measured at 5, 20 and 60 min after AGE stimulation. White bar, control; light grey bar, 25 μg/ml AGE; dark grey bar, 50 μg/ml AGE; black bar, 100 μg/ml AGE (n = 8, means ± SEM). Statistical analysis was performed by one-way ANOVA. *p < 0.05 vs control. The graph at 5 min is same as Fig. 2. Figure S4. AGE induced PF4 and 5-HT release from human platelets. Data were presented as means ± SEM (n = 8). Statistical analysis were performed by SPSS paired t test. White bars, control; black bars, AGE (100 μg/ml). *p < 0.05 vs control. Figure S5. AGE increased intracellular Ca2+ levels in platelets. Platelet was incubated with Oregon Green 488 (2 mM, Thermo Fisher) for 30 min and was washed with assay buffer (same as BDNF assay buffer). (a), Fluorescent intensity was measured by fluorescent microplate reader (Ex: 490 nm, EM: 530 nM) 3 min after AGE stimulation according to the manufacture’s instruction. Data are presented as means ± SEM (n = 30 (well)). Typical fluorescent photomicrographs of control (b) and AGE-treated (c) platelets. [file 12933_2017_505_MOESM1_ESM.pptx]

## Slide 1
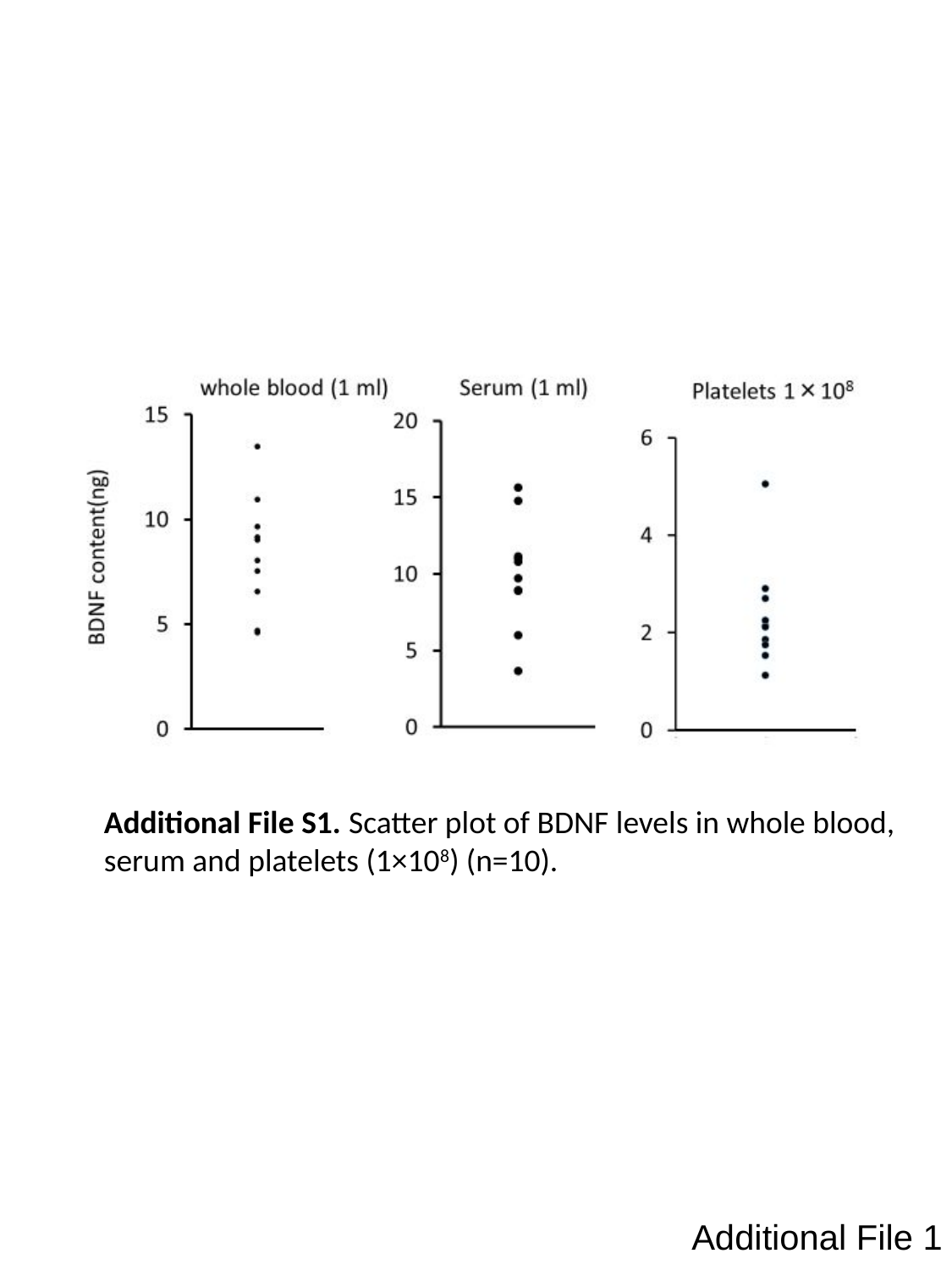

Additional File S1. Scatter plot of BDNF levels in whole blood, serum and platelets (1×10^8^) (n=10).
Additional File 1

## Slide 2
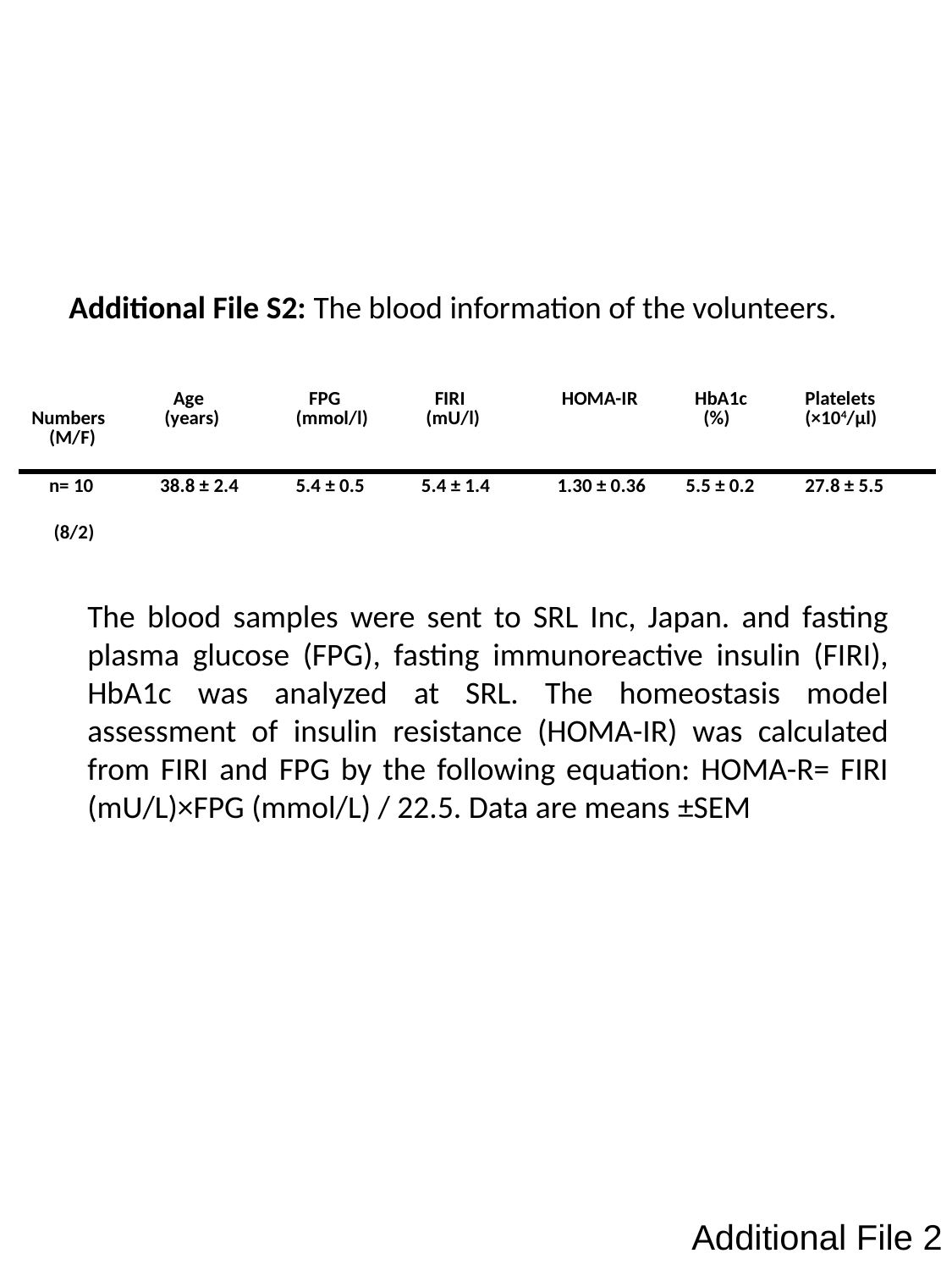

Additional File S2: The blood information of the volunteers.
| Numbers (M/F) | Age (years) | FPG (mmol/l) | FIRI (mU/l) | HOMA-IR | HbA1c (%) | Platelets (×104/μl) |
| --- | --- | --- | --- | --- | --- | --- |
| n= 10 　　 (8/2) | 38.8 ± 2.4 | 5.4 ± 0.5 | 5.4 ± 1.4 | 1.30 ± 0.36 | 5.5 ± 0.2 | 27.8 ± 5.5 |
The blood samples were sent to SRL Inc, Japan. and fasting plasma glucose (FPG), fasting immunoreactive insulin (FIRI), HbA1c was analyzed at SRL. The homeostasis model assessment of insulin resistance (HOMA-IR) was calculated from FIRI and FPG by the following equation: HOMA-R= FIRI (mU/L)×FPG (mmol/L) / 22.5. Data are means ±SEM
Additional File 2

## Slide 3
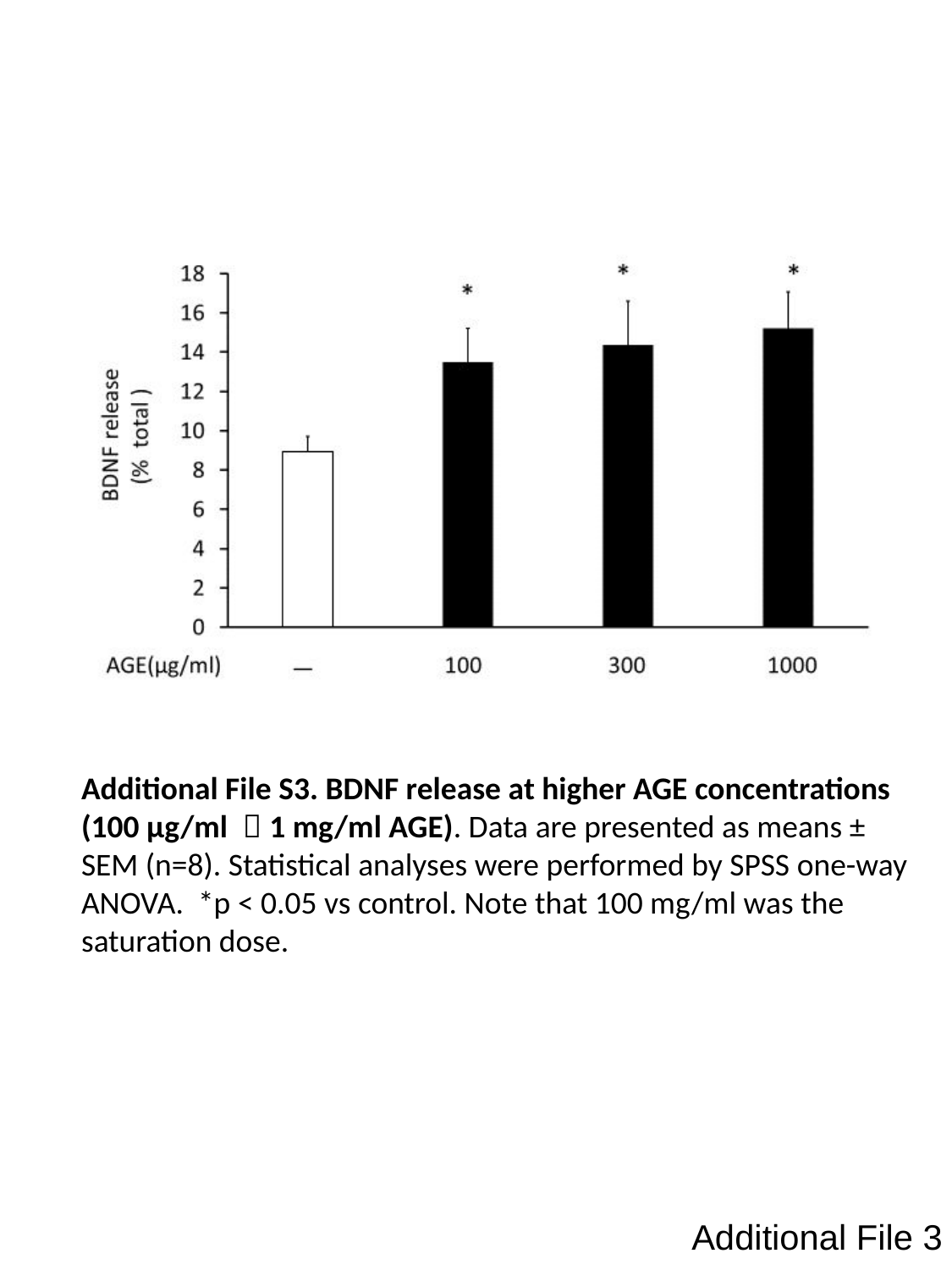

Additional File S3. BDNF release at higher AGE concentrations (100 μg/ml ～1 mg/ml AGE). Data are presented as means ± SEM (n=8). Statistical analyses were performed by SPSS one-way ANOVA. *p < 0.05 vs control. Note that 100 mg/ml was the saturation dose.
Additional File 3

## Slide 4
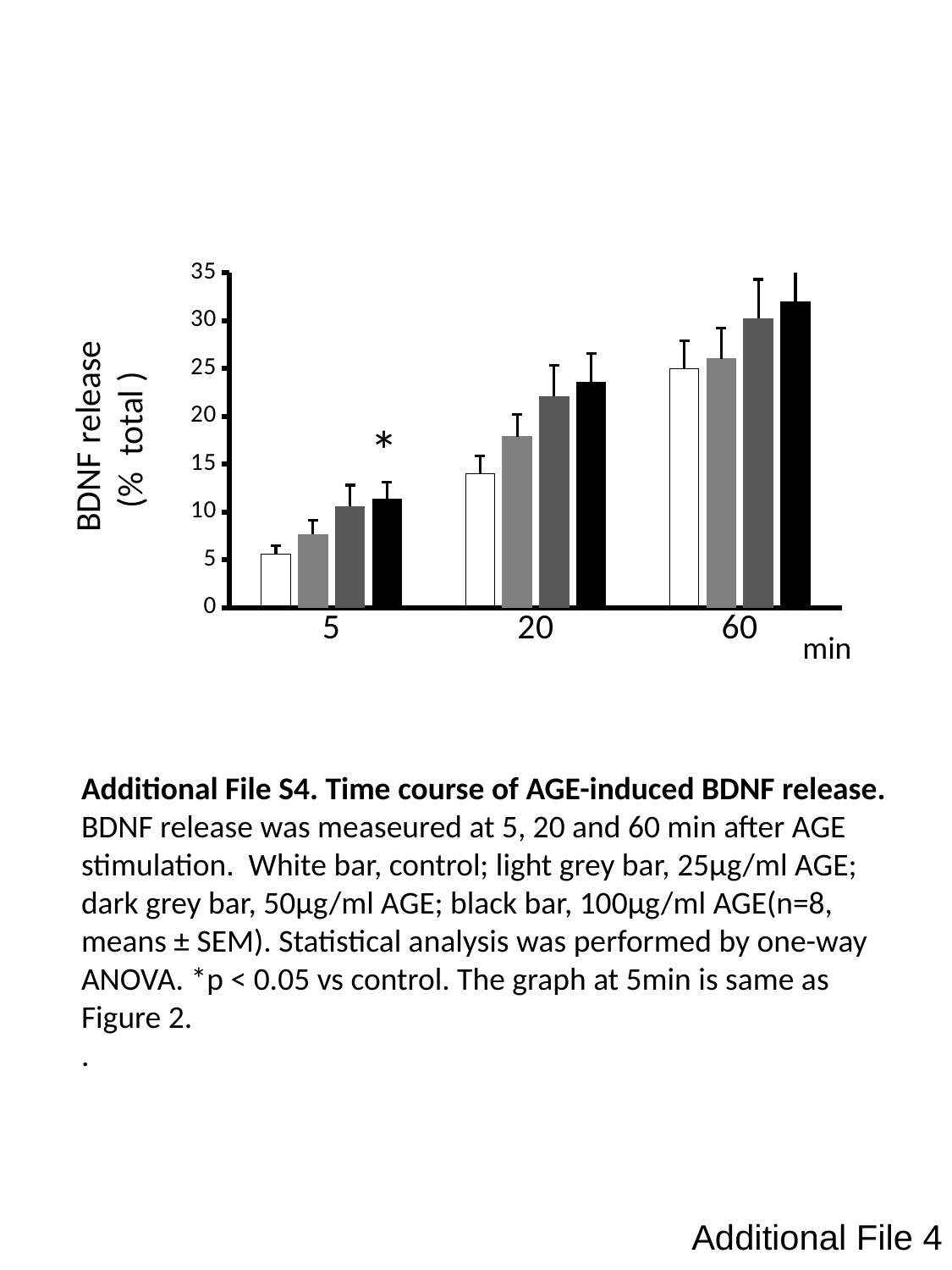

### Chart
| Category | con | AGE 25μg/ml | AGE 50μg/ml | AGE 100μg/ml |
|---|---|---|---|---|
| 5 | 5.615102891982095 | 7.654515552104452 | 10.56838741441663 | 11.343747404272019 |
| 20 | 14.00086363910757 | 17.856909029292385 | 22.06498637859243 | 23.575225690788248 |
| 60 | 24.9951911238172 | 26.019526193753926 | 30.225212487795815 | 31.922037408621176 | BDNF release
 (% total )
min
Additional File S4. Time course of AGE-induced BDNF release. BDNF release was measeured at 5, 20 and 60 min after AGE stimulation. White bar, control; light grey bar, 25μg/ml AGE; dark grey bar, 50μg/ml AGE; black bar, 100μg/ml AGE(n=8, means ± SEM). Statistical analysis was performed by one-way ANOVA. *p < 0.05 vs control. The graph at 5min is same as Figure 2.
.
Additional File 4

## Slide 5
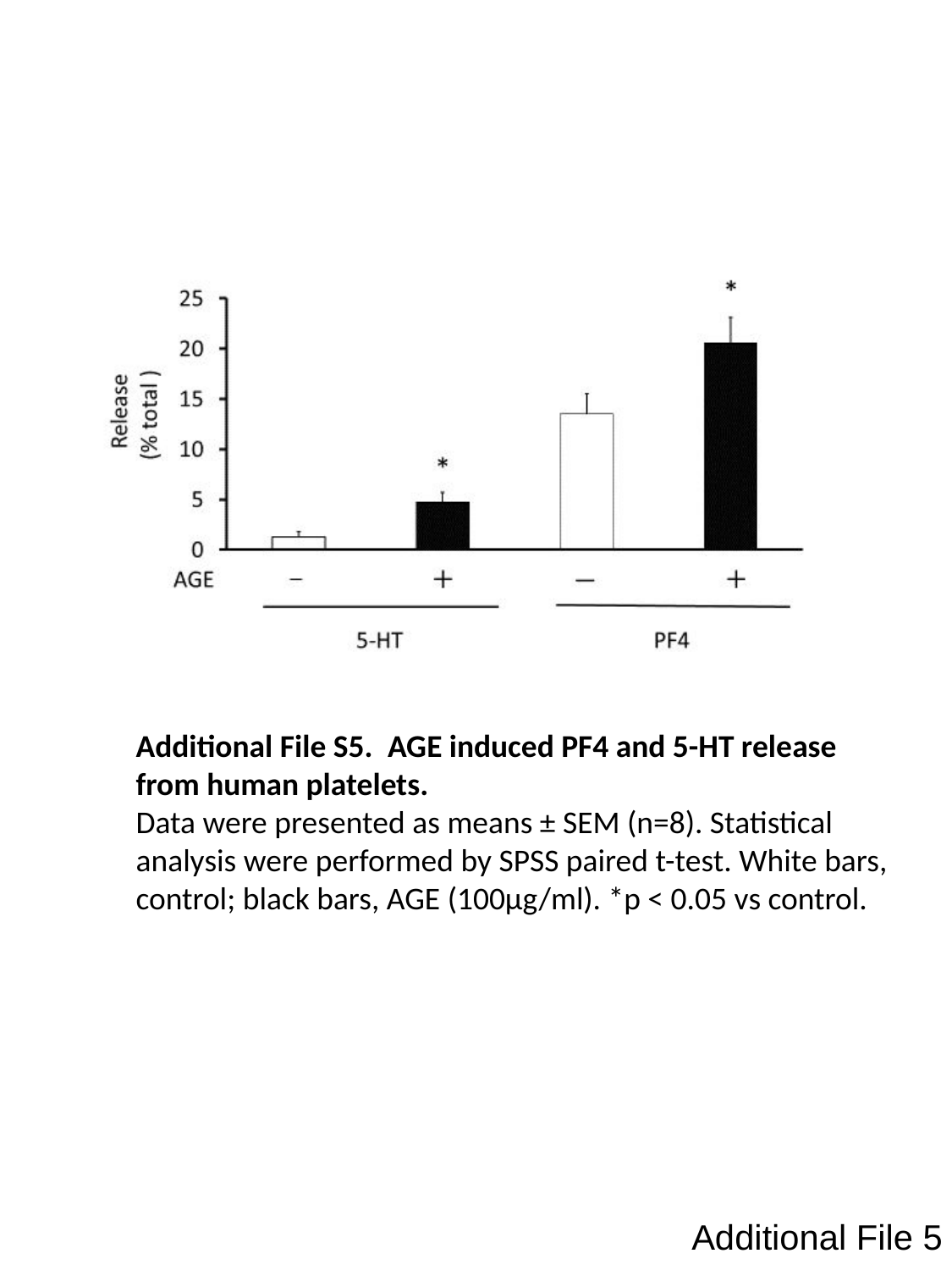

Additional File S5. AGE induced PF4 and 5-HT release from human platelets.
Data were presented as means ± SEM (n=8). Statistical analysis were performed by SPSS paired t-test. White bars, control; black bars, AGE (100μg/ml). *p < 0.05 vs control.
Additional File 5

## Slide 6
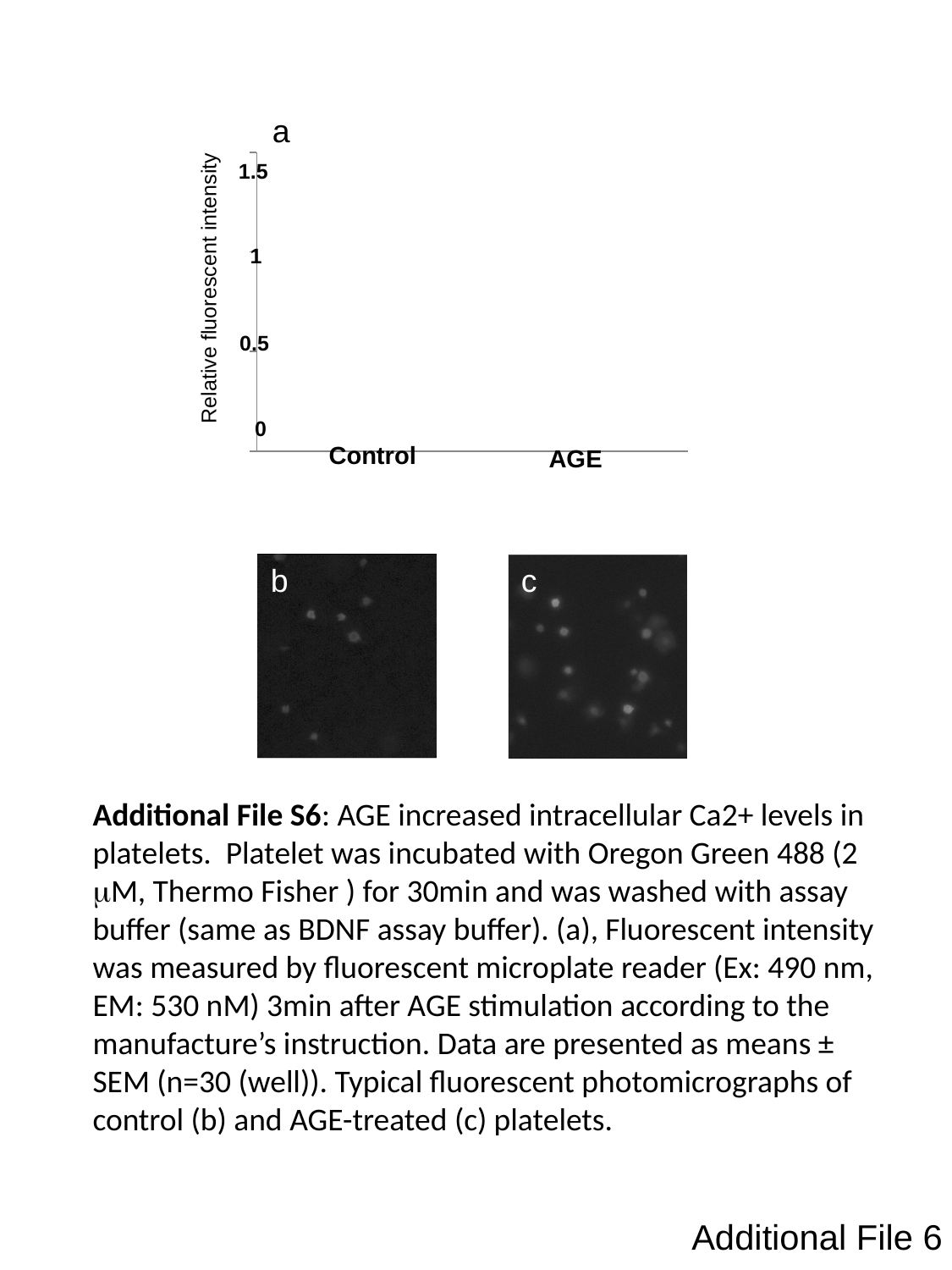

a
### Chart
| Category | |
|---|---|1.5
1
Relative fluorescent intensity
0.5
0
Control
AGE
b
c
Additional File S6: AGE increased intracellular Ca2+ levels in platelets. Platelet was incubated with Oregon Green 488 (2 mM, Thermo Fisher ) for 30min and was washed with assay buffer (same as BDNF assay buffer). (a), Fluorescent intensity was measured by fluorescent microplate reader (Ex: 490 nm, EM: 530 nM) 3min after AGE stimulation according to the manufacture’s instruction. Data are presented as means ± SEM (n=30 (well)). Typical fluorescent photomicrographs of control (b) and AGE-treated (c) platelets.
Additional File 6
